# Comparison of Representative Microservices Technologies in Terms of Performance for Use for Projects Based on Sensor Networks

**DOI:** 10.3390/s22207759

**Published:** 2022-10-13

**Authors:** Piotr Plecinski, Nataliia Bokla, Tamara Klymkovych, Mykhailo Melnyk, Wojciech Zabierowski

**Affiliations:** 1 Department of Microelectronic and Computer Science, Lodz University of Technology, ul. Wólczańska 221, 90–924 Łódź, Poland; 2 Department of Semiconductor and Optoelectronic Devices, Lodz University of Technology, ul. Wólczańska 211/215, 90-924 Łódź, Poland; 3 Department of Computer Aided Design Systems, Lviv Polytechnic National University, Mytropolyta Andreya St., 79013 Lviv, Ukraine

**Keywords:** Spring Boot, Micronaut, Quarkus, microservices, web application, sensors networks

## Abstract

Reading and analyzing data from sensors are crucial in many areas of life. IoT concepts and related issues are becoming more and more popular, but before we can process data and draw conclusions, we need to think about how to design an application. The most popular solutions today are microservices and monolithic architecture. In addition to this choice, there is also the question of the technology in which you will work. There are more and more of them on the market and in each of them it is practically possible to achieve similar results, but the difference lies in how quickly it will be possible and whether the approach invented will turn out to be the most optimal. Making the right decisions at the beginning of application development can determine its path to success or failure. The main goal of this article was to compare technologies used in applications based on microservice architecture. The preparation of a book lending system, whose server part was implemented in three different versions, each using a different type of technology, helped to achieve this goal. The compared solutions were: Spring Boot, Micronaut and Quarkus. The reason for this research was to investigate projects using sensor networks, ranging from telemedicine applications to extensive sensor networks collecting scientific data, or working in an environment with limited resources, e.g., with BLE or WIFI transmitters, where it is critical to supply energy to these transmitters. Therefore, the issue of efficiency and hence energy savings may be a key issue depending on the selected programming technology.

## 1. Introduction

One of the most important tasks before starting work on a project is to create an appropriate application architecture. The discussed issue has an impact on the functioning of the enterprise, the pace of changes, and security, among others. The consequences of this choice can be felt in everyday work, and changing the approach would involve long and costly changes that would cause a lot of problems and would slow down the development of applications from a business point of view.

In our department, we run many projects based on sensors or sensor networks. These include sensor network projects collecting data from complex high energy physics projects and large rotor machine endurance testing projects. Research related to smart clothing and telemedicine is also carried out. So far, in many situations, applications created by our teams have been executed in various programming technologies. Sometimes it was due to the technology being imposed by the project partner and sometimes simply because the language was better known or preferred in a given team. In addition to the general knowledge and experience of our programmers, which generally gave the knowledge of the best choice of programming technology, we found that this was a further field for optimization. This was especially so, since in some projects the processing time and communication speed could and did have a critical importance. For example, if data collected from sensors placed on the patient’s body sent data to a device and the device had to make a decision based on this analysis, time was of the essence. The delay in this case could mean the patient’s fall. In another project, the data collected using information from BLE beacons and WiFi transmitters, apart from the fact that they should be processed relatively quickly, additionally, due to the difficulties with powering sensors and beacons, it was crucial to minimize energy consumption, so it was necessary to reduce the transmission time. Yet in another project, due to problems with power supply in the form of a battery, it was necessary to reduce energy consumption in general, so it was necessary to optimize resource consumption, processing times, etc. so that the control process itself consumed as little of such valuable energy as possible. Therefore, although the problem of performance is known among other studies and other teams, the research they conducted did not prove useful to us. The tests that were planned and carried out by us have been tailored to our needs, including, among others, database operations. In the following, we refer to the state of art, pointing to possible gaps in the research conducted for our needs, without in any way detracting from the achievements of other scientists. We just needed something else.

## 2. Related Works

Considering current solutions, there are several aspects to consider, such as: efficiency, scalability, manageability, and reliability. Currently, the best-known solutions include monolithic architecture and microservices.

This topic is very popular nowadays and there are many new, fast-developing technologies that are constantly being researched and tested in order to find the best one to use.

Matthias Graf conducted a comparison of technologies used in microservices in terms of performance and ease of implementation. In performance tests, he looked at compile time and application startup, as well as peak performance. These are important factors, but there are many more aspects to consider when choosing a form of technology [1]. A more interesting and useful study was conducted by Roman Kudryashov, because, in addition to measuring the times of specific actions, he also paid attention to memory consumption, which is very important if we use cloud services [2]. Similar tests were recently conducted by a Polish team comparing the above-mentioned technologies with the Javalin framework, which is not a microservices solution and therefore was not included in our comparison. The tests traditionally checked the speed of specific actions and resource consumption [3]. The previously mentioned research focused on checking which technology was faster and consumed less resources, but did not address the issue of application stability under a heavy load or the speed of database operations. In production systems these aspects are crucial and determine customer satisfaction. Spring Boot is one of the most popular technologies and you can find many publications comparing it with others in terms of performance. An interesting study was conducted by Hardeep Kaur Dhalla. He tested Spring Boot’s capabilities in terms of load and resource consumption and compared it with the results of another well-known MS.NET technology. This was a very useful study, but unfortunately it did not include the research elements that are important to the developer, such as compilation time or test-run time [4]. The microservices architecture used by the authors is becoming more and more popular because it gives tremendous freedom in terms of technology choice. Aristide Massaga and Georges Edouard Kouamou are the authors of one of the most recent studies in this field. They decided to evaluate the adaptation of a given technology to microservices architecture. They approached the matter mathematically and developed a formula to present the results as a percentage. Unfortunately, they also focused on older technologies, such as Spring Boot and Java EE 7 [5].

Already in [6] they rightly note that the issue of the increasing use of microservices is worth examining. The authors tried to review the differences between the architecture of microservices and monolithic applications. They compared the performance of the monolithic application to the version in Consul and Euroreka when it comes to microservices. However, the developed comparison was not in-depth because, in principle, they focused only on the load test and concurrency testing. From our point of view, this is not enough. The authors [7] of the publication on Benchmarking for Microservices focused on the problem in a similar way. The fact of trying to answer the method of assessing the performance of this technology fits in with the demand for these results. Again, however, they did not address the key performance issues from our point of view. Our work presented in the article is a continuation of the problem of the performance of microservices for use in applications analyzing data from sensors. The issue of efficiency was worked on in relation to the solutions of the Amazon platform [8]. The authors [9] showed the efficiency of the applied microservice solutions in telemedicine with a cloud environment. They test their solution in a very general, and one can say comprehensive, perspective, i.e., the number of inquiries and the number of users. Although this is a very interesting and important study that gives an overview of the performance of the solution, we go further; that is, we consider the efficiency for atomic operations, and thus our research results provide a greater possibility to adapt the implementation of the sensor application, but also the design of the sensor network structure or even the returned data and their format. From this point of view, our research is more in-depth. The importance of using the sensor network in software via the microservices platform is also evidenced by its use in cattle breeding [10]. The authors of the publication in Applied Science [11] focused on the possibility and application of microservices for data exchange and production in industrial processes. Of course, it is no secret that microservices are suitable for such applications, but in this case, the authors were not limited by resources, as is often the case when analyzing data from a sensor network, when this analysis must be done quickly, and with the lowest consumption of resources and energy. Therefore, their research emphasizes the importance of the problem that was highlighted in this article. Finally, comparative performance studies can be found between monolithic and microservice architecture. Although the title [12] announces that it is a comparative study, it nevertheless focuses on examining general problems and benefits, and challenges in the transition from a monolithic to a microservice model. Although this is a very important voice in the discussion and contains important experimental data, they are, again, not related to the problem that can be encountered with the high requirements of applications that analyze ad hoc data from sensor networks, such as in medical applications, when the speed of data processing depends on the patient’s reaction or a decision about some action, while taking care to use the lowest possible consumption of energy and therefore also physical resources, including sensors in the network or communication beacons. The same is the case in [13], confirming the popularity of microservice solutions, but without any significant reflection on the efficiency of nuclear activities. In the applications discussed by these authors it may not matter, but they differ significantly from the environment we are considering. The authors of the next publication make an interesting comparison of the microservices technology of the most popular platform, but they still lack some of the tests that the authors have made in this article. Besides, the study conducted in [14] concerns only one of the platforms and there is a much narrower comparison than in this article because it concerns three technologies. In another study [15], the authors dealt with the tuning of microservice solutions, noticing the problem with matching performance to the needs. The authors analyzed the Bayesian optimization approach both in terms of solution speed and resource allocation efficiency. The results obtained by the authors do not exhaust the issues discussed in this article. The authors collected data from sensors [16] geographically distant from each other due to the essence of the implemented environment. Although we are dealing with a network of sensors, the performance issues that were at the basis of our research, in this case are not crucial and Panduman and others do not think about it at all in this respect. The authors analyzed [17] and collected runtime logs from six microservices in the retail domain. Although the study itself looks promising, it is quite superficial. It is an interesting approach in analyzing the impact of the communication protocol [18] used for microservices on the implementation performance. It shows that this has an impact on performance and resource consumption. The authors investigated and evaluated the microservice delivery performance of the selected platform [19] by considering the details of all layers in the modeling process. To this end, they first built a microservice platform on the Amazon EC2 cloud and then used it to develop a comprehensive performance model to perform conditioning analysis and capacity planning for large-scale microservice platforms. In other words, the proposed performance model provided a systematic approach to measuring the flexibility of a microservice platform by analyzing the provisioning performance on both the microservices platform and the back-end macro service infrastructure. From this point of view, this study is very useful, but it does not fit the microscale solutions that are so crucial for our tested solutions. The authors present the use of microservices [20] and their architecture from the sensor system and data processing using the developed environment. This demonstrates the effectiveness of this architecture for sensor network platforms.

Yilmaz and Buzluca [21] discussed the problem of maintaining microservices. In their work, they proved how important this aspect was in the maintenance of systems based on microservices and used fuzzy logic to do so. The topic of choosing the right microservice technology based on various frameworks, taking into account the response time, is discussed by Liu and his team [22]. This proved that this performance aspect of microservices should be taken into account in systems where processing time matters. Subsequent works deal in general with the design of microservice systems in order to ensure their best performance [23,24,25,26]. Another important aspect that appears in the literature and influences the design of sensor systems is security [27], in particular in edge computing, and the authors analyzed solutions based on AI.

It must also be said about the use of microservices in the production of similar ones to those we perform in our research. However, as you can see in these articles, the authors, although they show very interesting solutions, do not deal with performance in the way that we present. They treat microservices as a tool, without bending over details that could, or even certainly, affect the performance of the whole. First of all, we are talking about interesting works in the field of multisensory patient networks [28], very interesting work with the use of microservices for the measurement system “Nanoplasmonics” [29] or finally on the architecture of a system implementing multi-tenant vehicle monitoring [30].

The importance of the use of microservice solutions is demonstrated by, among others, Al-Debagy I Martinek and Mazlami with a team [31,32] proposing to migrate from a monolithic application to a microservices application. The methods and challenges in such a migration are also dealt with by Kyryk’s [33] team, focusing on the processes and methods of such conversion. The aspects of software engineering in such a conversion are dealt with by De Lauretis [34], taking these aspects into account, disregarding performance issues. A similar approach to the subject can also be found in other works and other groups [35,36,37]. This only confirms that the topic of migration from monolithic applications to microservices is on time, and therefore how many challenges, including performance challenges, are to be solved and described.

The importance of the performance aspect is understood by, among others, Mostof and his team [15], but also Wan and others [38], who focused on load balancing in microservice applications and Khazaei with his team who provided an analysis of provisioning in microservices [14]. The use of technology in IoT solutions in edge computing is a very interesting work by Nisansal [39]. They dealt with an important problem of efficiency, but the results of their research, although very interesting, do not correspond to our needs in specific design choices. However, it is a very important voice that such research and the perception of these problems is noticeable. The use of microservices in IoT is also a presentation of the design methodology, taking into account the challenges of IoT [40].

A very interesting work [41] related to the general comparison of monolithic applications and those based on microservices, but the authors focused on the aspects of performance and scalability. Although it is a very interesting work, from our point of view, it does not deal with detailed issues related to the problems that we encountered when creating applications for our projects with sensor networks. Herbke and his team [42] are heading towards optimizing solutions based on microservices by correctly identifying the need to analyze and optimize microservice solutions. The work by Li [43], which introduces the concept of quality in these solutions of microservice architecture, resounds in this vein.

Important selections are the works cited in succession [44,45,46,47,48]. In the first one we can find a comparison of monolithic and microservice architectures, also in terms of certain performance aspects, but to a certain degree of generality due to the selection of only one of the microservice platforms. The next one is managing resources in edge computing with the use of microservices. This is an interesting voice corresponding to the work presented in this article, but dealing with other aspects of this management, also not referring, as in our article, to various microservice platforms.

The next three articles (listed above in the citation) are an attempt to estimate the performance and microservice solutions in various approaches. It contains very useful research and results providing a lot of help in optimizing processes and implementation decisions. However, they differ significantly in their approach to ours. It does not mean that theirs is worse or ours is better. Our approach and research are simply a response to a specific problem in our teams’ research. Our results are a valuable answer for us and our projects, but also for other teams dealing with these problems. It is not an exaggeration theorem, because we obtained a positive reception of the results of our research through the exchange of experiences with other teams. The results of our research have often been met with positive curiosity and a positive response to the results and conclusions obtained in relation to various microservice platforms.

Nowadays, more and more institutions are using the cloud and that is why the authors thought it was worth checking and comparing currently available solutions. Micronaut and Quarkus are fairly young technologies and it is sometimes problematic to find research to help you decide which technology to choose for your project. Our study was conducted on three identical applications developed with three different technologies and was intended to extend the range of tests conducted so far and to identify the best application for the technologies.

## 3. Architecture

Many well-known companies have moved from monolithic architecture to microservices. One of the more famous examples is Netflix. The streaming service is connected to thousands of microservices and data store instances on AWS (Amazon Web Services) to serve millions of users around the world. The system is under heavy load due to tens of millions of real-time mutations, replicated globally, per second [49] and it still appears to be reliable.

### 3.1. Monolith

The term monolith refers to systems that are designed as single deployment units. Most often they have only one database, which is shared by all services. In theory, the biggest advantages of such architecture include its simplicity and accessibility. This approach does not require thinking about the division of services and functionalities, because everything takes place within one application. Unfortunately, in the case of an error occurring when even one service is called, the whole application usually stops working and finding the cause itself is often problematic. In such a central system, one technology is used and for some functionalities it is simply not advisable and in another one the given demand could be executed much faster and more efficiently. It is this lack of flexible approach to change and low scalability that are among the biggest arguments against this solution. The monolith is difficult to scale due to irregular consumption of different services deployed on a single application. When there is demand for highly consumed services, additional infrastructure is needed for the entire application, regardless of the increase in consumption of services. For this reason, sometimes server resources are wasted on unused services [50].

### 3.2. Microservices

The term microservices describes an approach to software development that decomposes business domain models into smaller and consistent services. An application consists of multiple modules and each module is responsible for a single functionality. Microservices are separate and fully autonomous components that communicate with each other to form a coherent whole. Usually, a separate team is responsible for the implementation of one such service. Such an approach enables independence in system development and does not impose the technology when implementing a given service [49].

Advantages:

- Scalability;
- Freedom of technology choice;
- Code quality;
- Stability;
- Shortening the production cycle.

Disadvantages:

- No transactivity;
- Challenging design of architecture;
- Unprofitable in small projects.

#### 3.2.1. Compared Technologies

In our study, we took the most popular, in our opinion, microservice technologies. We briefly outline them in the following sections.

#### 3.2.2. Spring Boot

When discussing the first technology, Spring Boot, it is important to mention Spring itself. It is a popular, open-source, JVM-based framework for creating standalone, production-grade applications [51]. Its creators tried to solve the problems appearing in Java Enterprise Edition (JEE). The initial versions were clumsy and the application development itself was not an easy or pleasant task. The solutions used in JEE were complex and not easy to configure. The goal of the developers was to create an accessible product for everyone and that is why they decided to provide default configurations at the start, which made the work much easier. One of the most important advantages of Spring is their own IoC container, which is responsible for creating, managing and configuring bean objects. It manages the entire life cycle from the *new* operator to the *finalize* method. The IoC abbreviation refers to the Inversion of Control pattern, which in practice means the transfer of responsibility for object creation to the Spring container. Referring to the topic, Spring Boot is a tool that makes developing web application and microservices with the Spring Framework faster and easier [52].

#### 3.2.3. Micronaut

It is a JVM-based technology that was created in 2018 and its creators are the people responsible for creating Grails. Micronaut is a modern framework for building modular and easily testable microservice applications. The supported programming languages are Java, Kotlin and Groovy [53].

The biggest differences between the Spring Boot and Micronaut include the method of bean creation. In Spring, during startup, classes with appropriate annotations are scanned, from which beans are then created. The described process is not the fastest one, because it is based on reflection. In Micronaut, a different approach was chosen. An annotation processor was used, which takes care of these issues at the compilation level. The consequence of such action is a shorter application startup time.

Micronaut was designed in such a way that it harmonizes with cloud solutions. It has built-in integration components for GCP (Google Cloud Platform) and AWS as well as for Kubernetes. Thanks to fast startup time and dependency injection during compilation, Micronaut is very well suited for serverless environments. Spring Boot suffers a bit more in this area because it uses more memory and takes more time than Micronaut, and the application customization itself is more difficult and requires more attention.

#### 3.2.4. Quarkus

RedHat is responsible for creating this technology and its first version was released in 2018. Quarkus was developed to work with the GraalVM universal virtual machine, but it is also possible to work with the JVM. The supported programming languages are Java and Kotlin. Quarkus does not use reflection, but injects dependencies at compile time. Thanks to that, the application starts up comparably as fast as Micronaut, and on native images startup times drop to the millisecond level. The presented technology was based on the Container First concept. It was created to have the best possible results in such categories as: memory consumption, runtime and docker image size. Such features have allowed it to become an effective platform for serverless, cloud and Kubernetes environments [54].

### 3.3. Popularity

The popularity of a given technology is worth noting, because when choosing technological solutions, the support of the community and the developers’ plans for the future should also be taken into account. The greater the popularity, the greater the chance that the authors of the project will develop it further. Another noteworthy factor is the fact that in case of encountering problems while programming, it is much easier to find help on the internet for technologies with a large number of supporters.

#### Github

When comparing the popularity of the analyzed technologies, it is worth looking at their code repositories on Github. The service provides the possibility to mark a given repository with a star, which means that a given user considers it noteworthy and is satisfied with the content provided.

**Figure 1:**
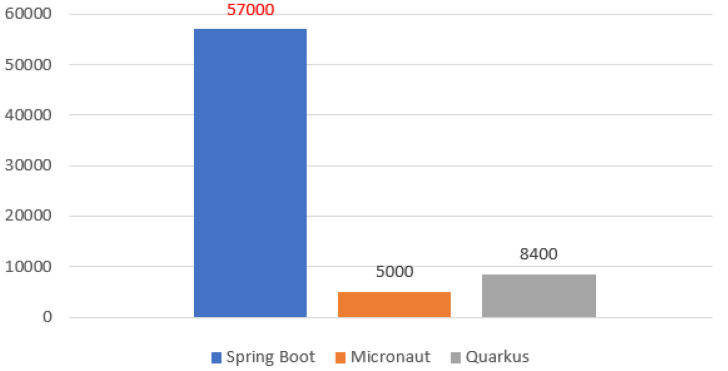
Popularity on Github.

Spring Boot has about 57,000 such ratings, Micronaut 5000, and Quarkus 8400 (Figure 1). These results confirm that the product from Spring is the most popular, which cannot be a surprise since it has definitely been on the market longer. As for Micronaut and Quarkus, they are newer offerings; Quarkus came a little later, and you can see that it is already more popular than Micronaut by more than 50%.

An interesting comparison was presented by the JAXenter portal [55], which in 2020 conducted a survey on popular technologies used for application development. Participants could indicate their attitude towards each of them on a scale from not interesting at all through to neutral and to very interesting.

**Figure 2:**
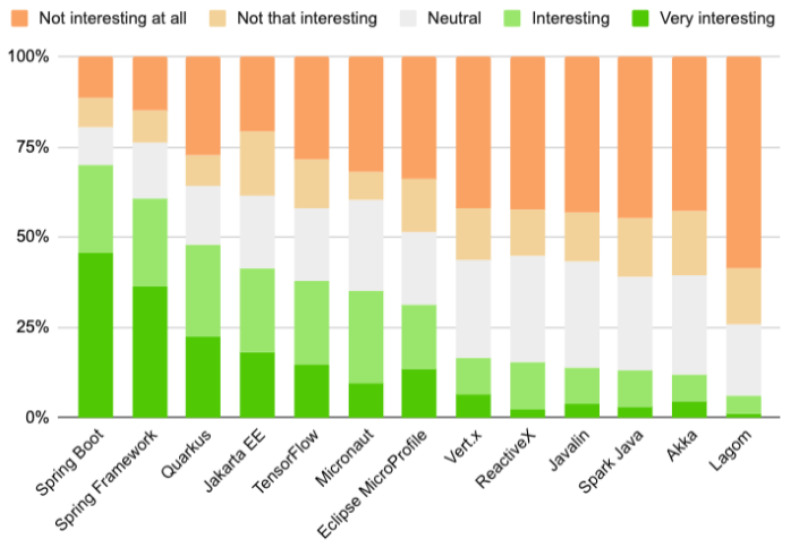
Results of a survey conducted by JAXenter [55].

Also in this comparison Spring Boot definitely wins, but again the result of Quarkus is worth noting. Despite the fact that it was created only in 2018, it already managed to take the third place. For 22% of respondents, it was very interesting, and for 25%, it was at least interesting. Micronaut, on the other hand, ranked in the middle (Figure 2).

## 4. Materials and Methods

Spring Boot (v. 2.3.10), Micronaut (v. 2.1.1) and Quarkus (v. 1.13.4) were tested for performance, stability and resource requirements. To test performance, three applications were developed that were as similar as possible. The projects were generated from the developers’ websites with only those dependencies that were necessary. The application itself was very simple. It consisted of a controller, a service and a class-containing bean configuration. The prepared service tests were written using RestAssuredMvc library, which allowed us to test API calls. This application will be called “First type app” in this article. One of the most important aspects of running the application in production is its stability, and to establish this stability, the three applications were implemented as a book rental service, which were based on a microservices architecture. That will be the “Second type app”.

The comparison was made under repeatable and identical conditions. In order to ensure real results, the tests were repeated serially, and the number of series was selected based on experience. So, for some tests, 5 was enough, and for others, as much as 1000 units. To increase the readability of the experiment, we first presented which tests were taken into account, and only later the results themselves, summarized in the form of graphs and with appropriate comments.

In our research work, equipment with the given specification was used. The presented tests took place on a laptop Lenovo Legion 5 15ARH05, whose parameters are as follows:

- CPU: AMD Ryzen™ 5 4600H 3.0–4.0 GHz;
- RAM: 16 GB;
- GPU: NVIDIA^®^ GeForce GTX™ 1650 + AMD Radeon™ Graphics;
- Drive: 512 GB SSD;
- OS: Windows 10 Home Edition.

Due to the prior analysis of the performance needs of our projects, only some aspects affecting performance were tested. These are the aspects that constitute a bottleneck or a potential field for optimization of the final application.

Due to the convenience and speed of development and deployment, we also took the compilation time into account.

The results obtained were the arithmetic average of five attempts for “First type app” The *mvn clean compile* command was used to compile.

Due to the specificity of the results, also for the remaining tests, we did not subject the obtained results to a special statistical evaluation, but only used the arithmetic mean. In our opinion, this is completely sufficient to obtain knowledge about the goal set at the beginning of the research. Entering complex and elaborate statistics would be an artificial activity.

Testing plays a very important role in our projects. In many situations, we cannot afford to run tests after the final application has been deployed.

Therefore, an important aspect that we took into account due to frequent changes in the application environment was the testing time.

The tests were written using RestAssuredMvc and checked the returned value, which was a simple String test. The presented result is the average of five trials for the “First type app”.

Due to possible restarts of the implemented system, sometimes the time of starting the application is of key importance. The result of the test is an average time needed to start the “First type app”, based on five attempts.

In all or almost all our projects, the applications work with the database. The time, efficiency of basic database operations is of great importance for the overall data processing and obtaining the appropriate system decisions. The result of the test is an average time needed to start the “First type app”, based on five attempts.

Therefore, the performance of save and read operations from the database was analyzed. Due to the high speed of the read operation, it was necessary to increase the number of cycles in order to obtain measurable results that could be compared.

For the “Save” operation, the result is the average of three attempts to add 1000 identical records to the database, and empty the *BOOKS* table.

On the other hand for the “Read” operation, the result of the test is an average time needed to read 10,000 identical records from the *BOOKS* table, based on three attempts.

Application stability is a very important aspect. Therefore, it is important to choose the technology with the greatest certainty of stability. For the same reason, C++ based systems do not fly into space.

Stability tests were conducted for the “Second type app”, using the popular tool Gatling. It allowed us to introduce an actor model oriented towards sending requests instead of creating threads and thus generating more workloads. One of its biggest advantages is the ability to create or record test scenarios. The test scenario used to check the application overload was recorded while using the developed library and included routine user actions, such as displaying data from the database.

Each technology was tested under identical initial conditions, where the number of actors was 50 and the database had, respectively, 500 users, 1000 reservations and 2000 books.

For each application, tests were performed to find the threshold of first errors.

A very important factor in our projects is the high volume of received data. Therefore, in the study, we took into account requests per second. For this kind of testing Apache Benchmark was used, which tested the exposed API with huge amounts of requests. The console command *ab -k -c 20 -n 10000 http://localhost:8081/test* was sufficient to run the test. The parameter *n* specifies the total number of http requests that were made. The *c* parameter specifies the number of clients that were created to send requests in parallel. *http://localhost:8081/test* is the address that was tested with parameters c = 20 and *n* = 10,000 respectively. The results obtained are shown in the graph below and are the average result from five attempts for the “Second type app”.

Of course, all the previously mentioned problems, whether related to energy management or response time, are influenced by the use of memory and processor—key resources. The Apache Benchmark tool presented in Section 4 was also useful for checking resource requirements. For each technology 20 such commands *ab -k -c 20 -n 10000 http://localhost:8081/test* were executed for each technology, and at this point the CPU and memory consumption was observed. VisualVM provides a way to see what is happening with applications running in a Java virtual machine. The initial results were not taken into account because the first queries are more for warming up the application.

## 5. Results

### 5.1. Compile Time

**Figure 3:**
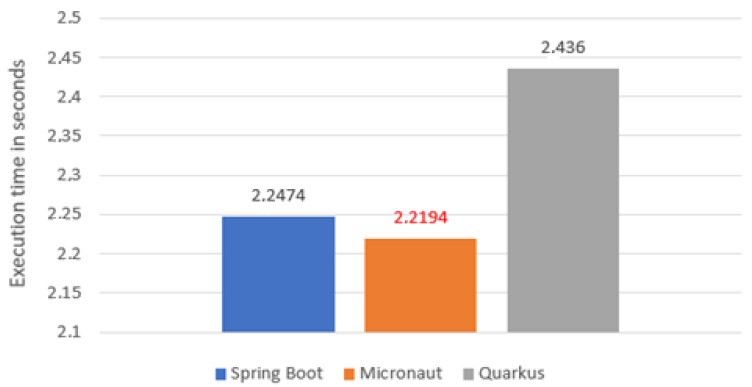
Average application compile time.

The first test focused on compile time (Figure 3). It was performed using *mvn clean compile* command. As you can see, the best time was achieved by Micronaut (2.2194 s), but its advantage over Spring Boot was minimal. Quarkus was slower than them by about 0.2 s.

The results of this test are not surprising, as one would have expected Spring Boot to be no worse than its competitors in this category. This is due to the fact that both Micronaut and Quarkus deal with bean issues at the compilation level, while Spring Boot only deals with them at runtime.

### 5.2. Test Time

**Figure 4:**
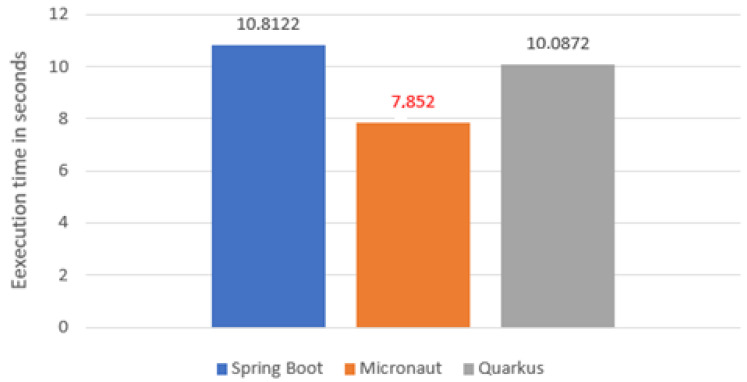
Average application test time.

The second aspect compared was test execution time (Figure 4). In this case, the Micronaut proved to be by far the fastest (7.852 s). Its result was by more than 2 s better than Quarkus, which was slightly ahead of Spring Boot. This may be due to the less complicated configuration of the class loader.

### 5.3. Startup of the Application

**Figure 5:**
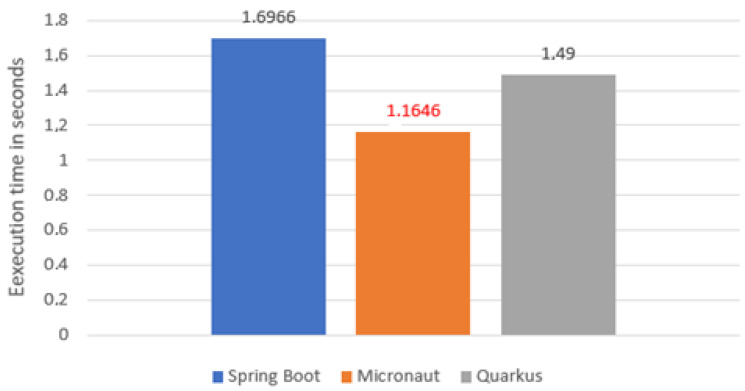
Average startup of the application time.

Next, the timing of one of the most important actions for a developer, which is launching the application, was examined (Figure 5). 

As mentioned earlier, Spring Boot scans annotated classes at startup from which it later creates beans, while the other technologies tested inject dependencies at compile time. So one would have guessed that in this comparison Spring Boot would turn out to be the slowest and that is exactly what happened, with Micronaut again being the fastest. This test would probably end up with a different result if the applications were run on native images, where Quarkus could present its full potential. According to some sources, its results then drop to the millisecond level.

### 5.4. Database Operations

#### 5.4.1. Save

**Figure 6:**
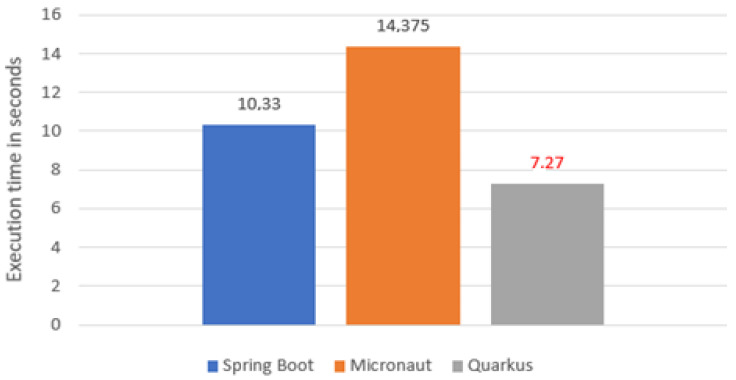
Average time of saving data to database.

Considering process of saving 1000 books (Figure 6), Quarkus proved to be the most efficient, with saving 50% better than Spring Boot and about twice as fast as Micronaut.

#### 5.4.2. Read

In the case of reading data, Quarkus was also the fastest, but in this case it had a decisive advantage over Spring Boot and was much slower than Micronaut (Table 1). When analyzing the results, it is worth noting that Quarkus uses PanacheRepository to manage data on the database, which is their own overlay on Hibernate. The developers’ goal was to create the simplest possible mechanism for communicating with the database. The results show that one of the biggest advantages of Quarkus is its speed, which makes it seem like an ideal candidate for native solutions.

### 5.5. Stability

#### 5.5.1. Tests for Identical Data

**Figure 7:**
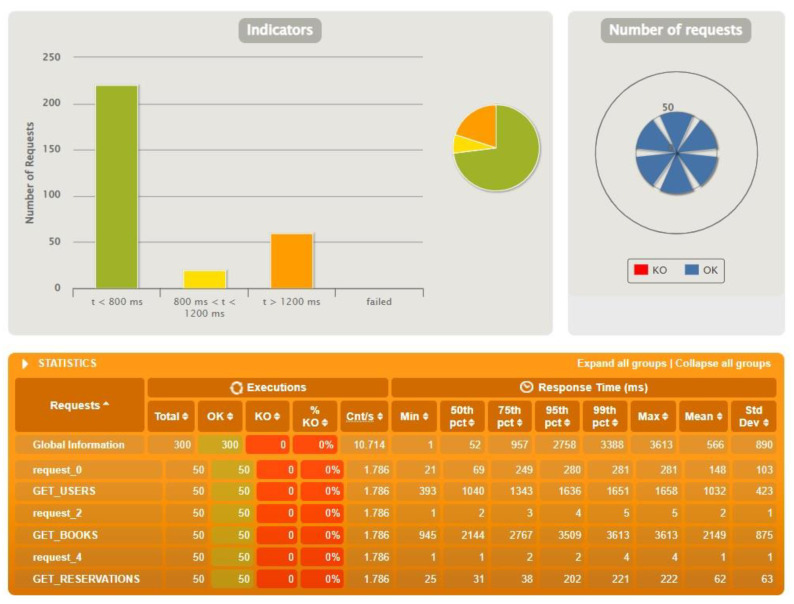
Results for test data: 500 users, 1000 reservations, 2000 books, 50 actors—Spring Boot.

**Figure 8:**
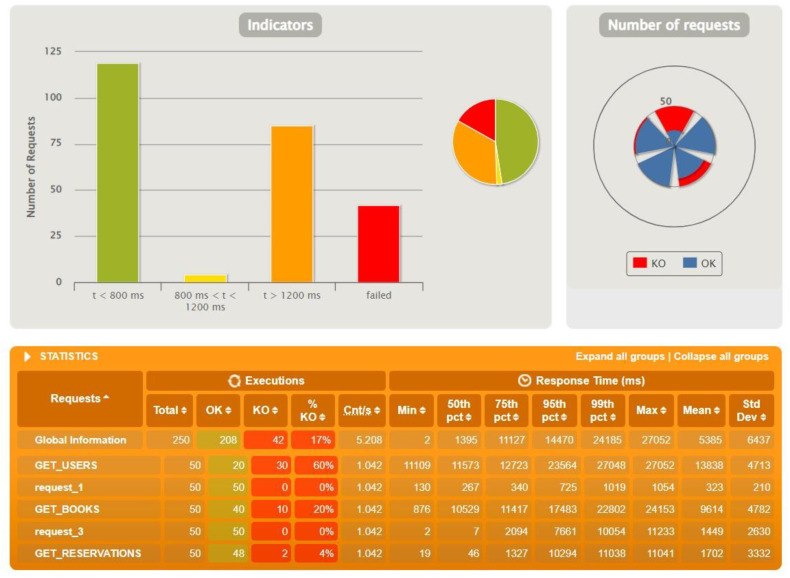
Results for test data: 500 users, 1000 reservations, 2000 books, 50 actors—Micronaut.

**Figure 9:**
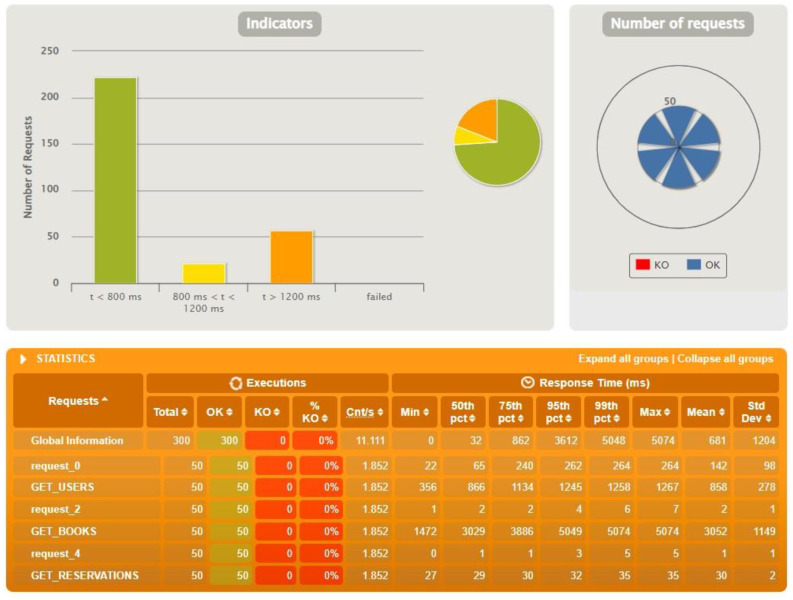
Results for test data: 500 users, 1000 reservations, 2000 books, 50 actors—Quarkus.

The following figure presents the results from the test run for Spring Boot (Figure 7). The presentation itself is detailed and easy to read for the user. As you can see, Spring Boot handled this test without any major problems. Analyzing the figure, you can see the division into three parts. In the upper left corner, there is a bar chart that represents how many queries were executed in under 800 ms, how many in the 800–1200 ms range, and how many in over 1200 ms. The fourth bar reports errors, but none appeared here. In the upper right corner is a slightly modified pie chart where you can see how many queries of a given type were correct. In the case of Spring Boot, they all executed and there were no KOs. Below the graphs are more detailed statistics, visualized by the previously mentioned charts. Below are also the results for Micronaut (Figure 8) and Quarkus (Figure 9).

The figures above (Figure 7, Figure 8 and Figure 9) show the results of tests conducted using Gatling for the three technologies for an identical test scenario. The database had 500 users, 1000 reservations and 2000 books. The number of actors in the script was set to 50. The interesting requests sent from the application were those related to users, reservations and books. The abbreviations were taken from the view representing the data by Gatling. As can be seen, Spring Boot and Quarkus handled this task with 100% efficiency. Micronaut’s results, on the other hand, are already much worse in comparison. In this case, 42 queries failed. Analyzing the logs received in the Gateway microservice console, we were able to determine that the errors were caused by exceeding the default time limit.

#### 5.5.2. Achieved Limits

**Figure 10:**
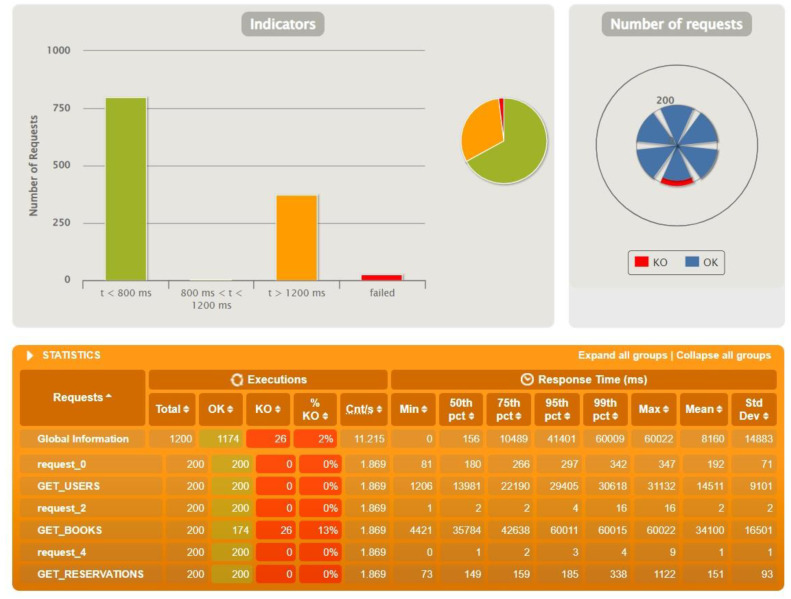
Results for test data: 2000 users, 4000 reservations, 8000 books, 200 actors—Spring Boot.

**Figure 11:**
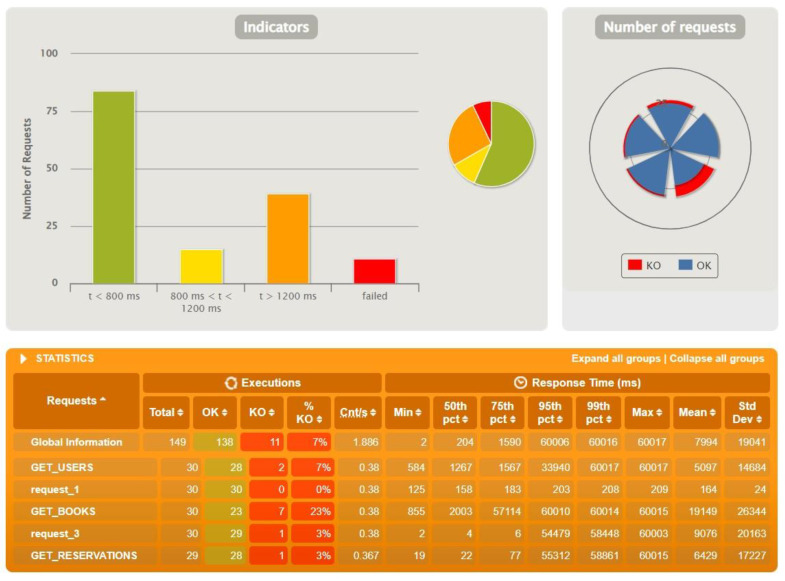
Results for test data: 500 users, 1000 reservations, 2000 books, 30 actors—Micronaut.

**Figure 12:**
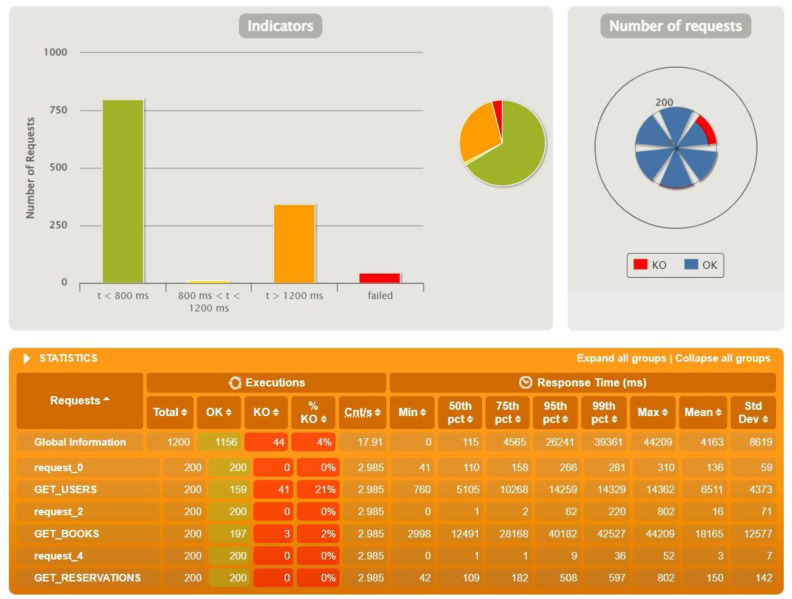
Results for test data: 1000 users, 2000 reservations, 4000 books, 200 actors—Quarkus.

After the first tests with identical test data, further tests were performed to find the threshold of occurrence of the first errors. During the tests the resources stored on the database and the number of actors were increased accordingly. The achieved limits are presented in the following graphs—Figure 10 for Sping Boot, Figure 11 for Micronaut and Figure 12 for Quarkus.

In the load test (Table 2), the clear winner was Spring Boot. The first problems started to appear when the number of actors was equal to 200 and the number of records stored on the database was as follows: 2000 users, 4000 reservations and 8000 books. The cause of the errors was the default timeout set to 60,000 ms.

With Quarkus, the first failed queries appeared with 200 active actors and twice as little data stored in the database as with Spring. Application logs allowed us to discover the cause of the problems and it was a broken connection to the database.

Micronaut at the initial test already noted problems with exceeding the time threshold on the gateway, so here we had to look for limits by reducing the parameters. For the initial data stored on the base and with 50 actors, significant problems were noted, but when reducing the latter value to 30, the situation improved significantly, and it can be considered that the maximum values for Micronaut oscillate within these limits.

To sum up, Gatling turned out to be a great and simple tool for application overload testing. Created test scenarios allowed us to mimic real user traffic as it occurs in systems already running on the client’s production environment. The winner of the test was Spring Boot. It maintained stability and responded to queries for the longest time, and the problems encountered were caused by the timeout set at 60,000 ms. It was followed by Quarkus, which also started to report the first irregularities while handling 200 actors, but twice as little data. The most surprising results concern Micronaut, because it achieved much worse results than the rest. The reasons for such results can be found in the default configuration of each technology. The main reason for the errors was exceeding the time limit or breaking the connection with the database. Potentially after adjusting the configuration to the application architecture, the results could look different.

### 5.6. Request per Second

**Figure 13:**
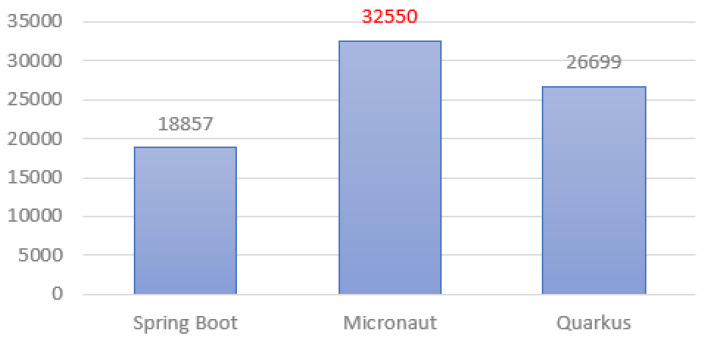
Average amount of requests per second.

In this test (Figure 13), Micronaut proved to be the best, achieving a result about 20% better than Quarkus and almost twice as good as Spring Boot.

### 5.7. CPU and Memory Usage

**Figure 14:**
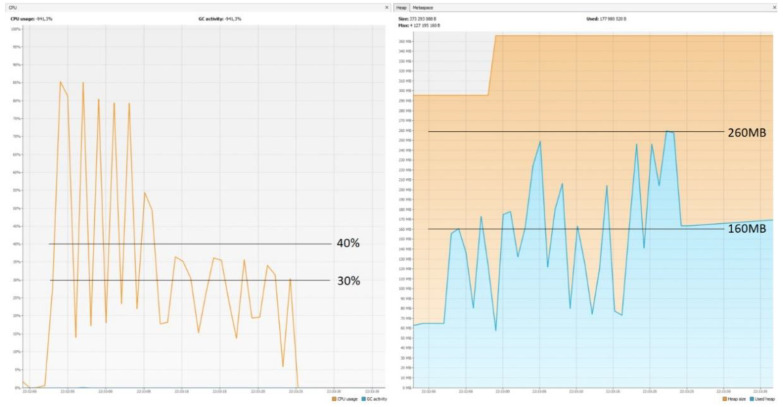
CPU usage: 30–40%; memory usage: 160–260 MB—Spring Boot.

**Figure 15:**
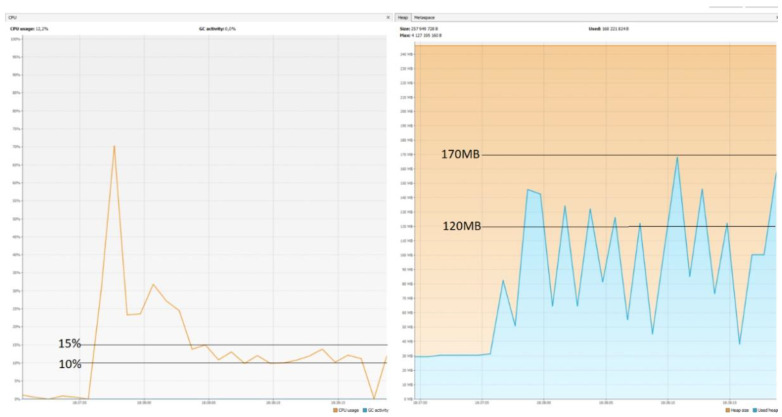
CPU usage: 10–15%; memory usage: 120–170 MB—Micronaut.

**Figure 16:**
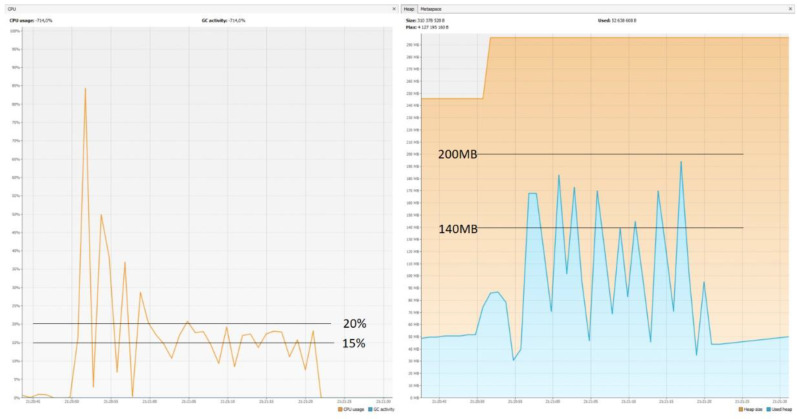
CPU usage: 15–20%; memory usage: 140–200 MB—Quarkus.

The graphs (Figure 14, Figure 15 and Figure 16) show that Micronaut consumed the least resources. Quarkus was slightly worse, but its results in comparison with those achieved by Spring Boot were still much better. These results confirm the trend that newer technologies are geared towards running in serverless environments. They are designed to keep startup time as short as possible and memory consumption as low as possible, since charges are incurred for the direct execution time of given functions. One of the reasons Spring consumes so much memory is the already mentioned reflection mechanism, which is not ideal when it comes to optimization.

## 6. Discussion

At the beginning it is worth noting that in each of these technologies it was possible to implement the assumed functionalities in the test application and the way of implementation looked similar everywhere. The biggest differences could be seen in the used annotations and in the configuration issues. From the perspective of the authors, who had the most experience with Spring Boot, getting started with the other technologies was not too difficult because the developers provide project generators and support the developer with rich documentation.

What turned out to be problematic was finding solutions when errors occurred in the application. Spring Boot is a few years older than the other two items, and because of that it was much easier to find help on the Internet. It is more popular and has decidedly more community support at this point, but in a few years, this should change in favor of Micronaut and Quarkus, as they have a lot to offer.

The tests conducted have shown that Spring Boot’s younger rivals perform better in several key elements, such as application startup time and resource consumption. This is due to the fact that dependencies are injected at the compilation level, which helps to speed up the process. However, when tested for application robustness to overloads, Spring Boot proved to be the more stable solution.

From the analysis of all these results, a very important point emerges. It is true that some results could be expected, but it is one thing to suppose and another to prove, and this is the role of the research process. Most of the results, however, were not at all obvious and both our own tests and the tools used have shown the real features of each technology.

## 7. Conclusions

When choosing the right technology for a project with microservices and lots of sensor data, you need to consider what it will entail. If you intend to run in the cloud, it is better to use Micronaut or Quarkus, as they are designed to run in the cloud, where costs are incurred based on time of use and resources consumed. For server-side solutions, the proven Spring Boot may be a more efficient choice.

Our research showed very important information related to the performance of each of the tested technologies, when used precisely for applications related to sensor networks. Of course, the conclusions drawn from the research are applicable to each application written in the technologies mentioned, but most importantly, the specificity of tests selected in terms of the challenges of our projects has proved that the choice of technology is not arbitrary. When developing applications on the edge of performance or with limited resources, the conclusions of our research can and are crucial.

Our work allowed for better selection of technologies in terms of various requirements in our projects. Each project has different requirements, ranging from efficiency, speed and energy consumption. The presented study enables such a selection of technologies to optimize certain aspects of our projects based on microservices. These are projects ranging from small networks based on sensors used in telemedicine to large networks of sensors used in industry. The authors plan further research in this area in order to check other microservice platforms and their suitability for our research. The conducted research is in line with the research carried out by our team to research the efficiency of various pro-programming technologies and programming languages and to use them to optimize the systems we design. The results of these studies will certainly be useful and are now useful for other teams, for which the authors already had information.

The results of our research are applicable in our work on the projects mentioned in the introduction to this article, and also for future implementations. We believe that the results and conclusions obtained as a result of this study will be useful also for others, due to the universality of this comparison, which certainly fills a certain gap in information on this type of problem.

## Figures and Tables

**Table 1:** Average time of reading and writing data to/from database.

	Spring Boot [s]	Micronaut [s]	Quarkus [s]
Write (1000 cycles)	10.330	14.375	7.270
Read (10,000 cycles)	0.152333	2.665000	0.024817

**Table 2:** Load limits for tested technologies.

	Spring Boot [s]	Micronaut [s]	Quarkus [s]
Database load	2000 users	500 users	1000 users
	4000 reservations	1000 reservations	2000 reservations
	8000 items	2000 items	4000 items
Actors	200	30	200
OK requests	574	30	200
KO requests	26	11	34

Successful amount for queries is OK, and for invalid KO. The abbreviations have been taken from the Gatling data view.

## Data Availability

Not applicable.
